# The Fold Variant BM4 Is Beneficial in a Therapeutic Bet v 1 Mouse Model

**DOI:** 10.1155/2013/832404

**Published:** 2013-09-23

**Authors:** Ulrike Pichler, Claudia Asam, Richard Weiss, Almedina Isakovic, Michael Hauser, Peter Briza, Fatima Ferreira, Michael Wallner

**Affiliations:** ^1^Christian Doppler Laboratory for Allergy Diagnosis and Therapy, University of Salzburg, 5020 Salzburg, Austria; ^2^Department of Molecular Biology, University of Salzburg, 5020 Salzburg, Austria

## Abstract

*Background*. Specific immunotherapy using recombinant allergens is clinically effective; still wild-type allergens can provoke treatment-induced side effects and often show poor immunogenicity *in vivo*. Thus, we tested the low IgE-binding, highly immunogenic fold variant BM4 in a Bet v 1 mouse model. *Methods*. Recombinant BM4 was used as active vaccine ingredient to treat mice sensitized to Bet v 1. As controls, mice were treated with either Bet v 1 or sham, and the humoral as well as cellular immune response was monitored. Moreover, lung function and lung inflammation were analysed. *Results*. BM4 was more effective than wild-type Bet v 1 in inducing Bet v 1-specific blocking antibodies as well as IFN-**γ** and IL-10 producing T cells. Further, birch pollen induced lung inflammation could be ameliorated significantly by BM4 treatment as demonstrated by a reduction of airway hyperresponsiveness and drastically decreased eosinophil counts in bronchoalveolar lavage fluids. *Conclusion*. The study outlines the high potential of BM4 as vaccine candidate for the treatment of Bet v 1-mediated birch pollen allergies.

## 1. Introduction 

Pauli et al. impressively demonstrated in a multicenter, randomized, double-blind placebo controlled trial that recombinant Bet v 1 can effectively replace birch pollen extracts in specific immunotherapy (SIT) of birch pollen allergy [1]. Nevertheless, the treatment of subcutaneous SIT is cumbersome, and treatment-induced local adverse reactions were reported during the trial. Thus, low IgE-binding recombinant allergens or derivatives thereof, which exhibit enhanced immunogenicity, will provide a groundbreaking alternative to wild-type allergens. Such molecules can be engineered to address the innate immune system to effectively redirect the TH2 immune response inherently activated by allergens. As previously published, a fold variant of Bet v 1.0101 (termed Bet v 1 thereafter), BM4, is suggested to improve efficacy of SIT. Whereas recombinant Bet v 1 induces primarily a TH2 biased immune response, BM4 is able to skew the immune response towards TH1 [2, 3]. To investigate the effects of BM4 as novel therapeutic, we compared the impact of recombinant Bet v 1 and, its fold variant BM4 in a therapeutic mouse model.

## 2. Material and Methods

### 2.1. Treatment Model

**Figure 1:**
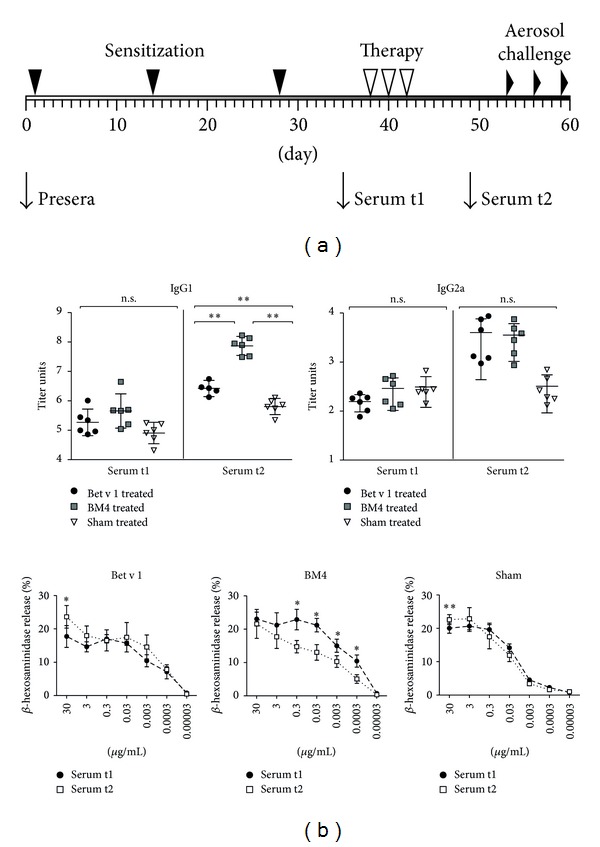
(a) Schematic representation of the therapeutic mouse model. (b) BALB/c mice were treated with either Bet v 1 (circles), BM4 (squares), or sham (triangles). Bet v 1-specific IgG levels were determined by ELISA, IgE by mediator release assays. Means ± SD are indicated, *P*-values were calculated with *t*-tests and paired-samples *t*-test, respectively (*P < 0.05, **P < 0.01).

8- to 10-week-old female BALB/c mice were purchased from Charles River Laboratories (Sulzfeld, Germany) and used for experiments 4 days after arrival (treatment schedule as shown in Figure 1(a)). All animal experiments were conducted according to the guidelines of the Austrian Ministry of Science (BMWF-66.012/0011-II/10b/2010). Six mice per group were sensitized subcutaneously (s.c.) with 5 *μ*g Bet v 1 adsorbed to Alugel-S (Serva, Heidelberg, Germany) bilaterally in the lumbar region, followed by three intraperitoneal (i.p.) injections of 25 *μ*g Bet v 1, or BM4 in PBS, or PBS alone. Aerosol challenges were performed with nebulized birch pollen extract (10 mg in 10 mL PBS) to induce airway hyperresponsiveness (AHR). ELISA and mediator release assays with murine sera were performed as described [2, 4]. In brief, for ELISA NUNC Maxisorp plates (Thermo Fisher Scientific, Waltham, USA) were coated with 2 *μ*g/mL antigen solution over night at 4°C. Murine sera were applied in serial dilutions and incubated for 2 h at room temperature. Bound antibodies were detected with appropriate alkaline phosphatase-conjugated secondary antibodies (all from SouthernBiotech, Birmingham, USA) followed by chromogenic substrate development. Mediator release assays were performed using the cell line RBL-2H3 (ATCC number CRL-2256) passively sensitized with murine immune sera. After removal of the sera, antigen was added in serial dilutions and mediator release was determined by enzymatic cleavage of the fluorogenic substrate 4-methyl umbelliferyl-N-acetyl-*β*-glucosaminide (Sigma Aldrich, St. Louis, USA). *β*-hexosaminidase release is expressed as a percentage of the total enzyme content of Triton X-100-treated RBL-2H3 cells. All experiments were performed in duplicates. 

For determination of AHR, mice were anesthetized, intubated, and ventilated using FinePoint apparatus (Buxco, Wilmington, USA). After baseline measurements mice were exposed to nebulized PBS, followed by challenge with increasing concentrations of nebulized methacholine (5–20 mg/mL, Sigma, UK). For determination of lung eosinophilia, cells in bronchoalveolar lavage (BAL) fluids were stained with CD45-APC (Immunotools, Friesoythe, Germany), Siglec-F-PE (BD Biosciences, San Jose, USA), CD4-FITC (BioLegend, San Diego, USA), and CD19-APC-Cy7 (eBioscience, San Diego, USA). Cells were measured on a FACS Canto II flow cytometer (BD Biosciences); data analysis was performed using FACS DIVA software (BD Biosciences). IL-5 in BAL fluids was quantified using a ready-set-go ELISA (eBioscience). Supernatants of restimulated splenocyte cultures were analyzed using mouse TH1/TH2/TH17/TH22 13plex FlowCytomix kit (eBioscience). Data were analyzed with the FlowCytomix Pro software (eBioscience). Statistical analyses were performed with *t*-tests or paired-samples *t*-tests, respectively. *P*-values <0.05 were considered statistically significant. 

## 3. Results 

### 3.1. BM4 Treatment Induced Cross-Reactive IgG Antibodies Whereas Bet v 1-Specific IgE Was Reduced

BM4 treatment induced 122 times higher levels of Bet v 1-specific IgG1 in sensitized animals, whereas Bet v 1 treatment resulted in 4 times elevated IgG1 levels compared to sham treated animals (Figure 1(b)). Treatment with either antigen led to an increase of IgG2a (BM4 treatment 12 times and Bet v 1 treatment 5 times elevated IgG2a compared to sham). To determine the levels of Bet v 1-specific IgE before and after SIT treatment, mediator release assays were performed. RBL-2H3 cells were passively sensitized with murine sera, and after removal of unbound antibodies mediator release was triggered by the addition of Bet v 1. We found no significant changes in mediator release before and after SIT using sera of animals, which have received either Bet v 1 or sham treatment. However, BM4 treatment was able to significantly reduce biologically functional Bet v 1-specific IgE levels (Figure 1(b)). 

### 3.2. BM4 Treatment Effectively Ameliorated Birch Pollen Induced Lung Inflammation

**Figure 2:**
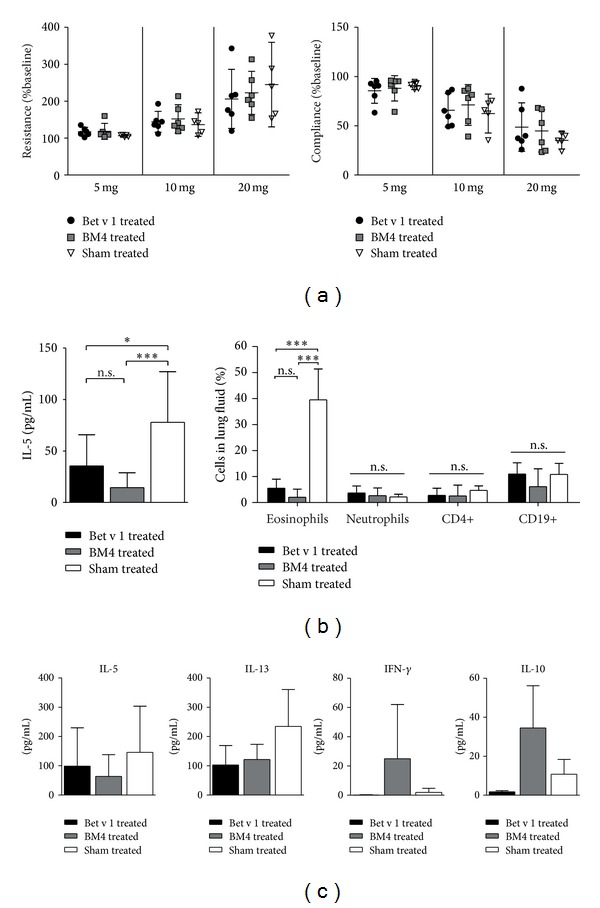
(a) Lung resistance and compliance of treated BALB/c mice were determined upon challenge with increasing levels of methacholine and are shown as deviation from baseline. (b) In BAL fluids IL-5 levels were determined by ELISA and lung infiltration by flow cytometry. (c) Cytokines secreted by re-stimulated splenocytes were quantified by FlowCytomix assay. Data are expressed as mean ± SD; statistics were calculated by *t*-test (*P < 0.05, **P < 0.01, ***P < 0.001).

Lung functions of animals treated with Bet v 1, BM4, or sham were determined after birch pollen aerosol challenge. We found a trend that allergy treatment with BM4 as well as Bet v 1 was able to improve lung functions after methacholine challenge compared to sham (Figure 2(a)). BAL fluids of animals treated with BM4 showed an eosinophil reduction of 95%, in parallel with an 82% reduction of IL-5, whereas Bet v 1 treatment resulted only in 86% reduction of eosinophils and 54% reduction of IL-5 compared to sham treated animals (both are shown in Figure 2(b)). 

### 3.3. Treatment with BM4 Was Able to Shift an Allergic Immune Response

Analyses of splenocyte supernatants of either actively or sham treated animals, which were re-stimulated with Bet v 1, revealed a clear trend showing that BM4 treatment led to suppression of the TH2 cytokines IL-5 and IL-13 accompanied by an upregulation of IFN-*γ* and IL-10. Compared to sham treatment suppression of IL-5 and IL-13 was also observed in the Bet v 1 treated animals; however Bet v 1 therapy failed to induce key cytokines of either TH1 or Treg cells (Figure 2(c)).

## 4. Discussion

As previously reported, a genetically modified variant of Bet v 1, BM4, showed reduced binding of human serum IgE from birch pollen allergic donors as well as increased T cell activating properties. This was a result of an enhanced activation of dendritic cells by BM4, a property directly linked to the structural alteration of the molecule [2, 3]. To evaluate the therapeutic potential of BM4 *in vivo* we established a mouse model of birch SIT. Since Bet v 1 is considered the main sensitizing agent in birch pollen showing a sensitization frequency of >95% [5], mice were immunized with Bet v 1 and thereafter treated with Bet v 1, BM4, or sham. Active treatment with BM4 induced significantly increased levels of Bet v 1-specific IgG1 antibodies compared to Bet v 1 or sham. IgG2a was also increased, though the results were not significant. To analyze the IgE levels of Bet v 1 before and after treatment, mediator release assays were performed. This allowed determining the IgE response without interference of serum-derived IgG antibodies. Of note is that only BM4 treatment reduced Bet v 1-specific IgE significantly. To compare the lung condition of treated versus untreated animals invasive measurements of the pulmonary function as well as analyses of BAL fluids were performed. By determining AHR we found a trend showing the improvement of lung function after active treatment compared to sham; moreover the eosinophil counts as well as IL-5 levels in BAL fluids were significantly reduced. Thus, we conclude that treatment with either Bet v 1 or BM4 could improve the lung functions of Bet v 1-sensitized animals compared to PBS. In splenocyte cultures, BM4 treatment led to the induction of the allergy-suppressing cytokines IL-10 and IFN-*γ*, accompanied by a suppression of IL-5 and IL-13 levels. The latter was also observed for Bet v 1 treated animals; however Bet v 1 failed to induce IFN-*γ* and IL-10 production. Though not statistically significant, this trend is an indication for altered immune polarizing properties of BM4, which is supported by previously published data on the molecule [2].

## 5. Conclusion

The increased immunogenicity of BM4 in combination with its immune polarizing property will allow a dose reduction in SIT without sacrificing efficacy. BM4 was shown to effectively induce IgG antibodies cross-reactive with wild-type Bet v 1 paralleled by a suppression of Bet v 1-specific IgE. Moreover, T cells primed during BM4 treatment produced antiallergic cytokines upon Bet v 1 restimulation. The study outlines the high potential of the molecule as vaccine candidate and encourages clinical application.
